# Feasibility and effectiveness of a novel dynamic arm support in persons with spinal muscular atrophy and duchenne muscular dystrophy

**DOI:** 10.1186/s12984-021-00868-6

**Published:** 2021-05-21

**Authors:** Mariska M. H. P. Janssen, Jolinda Horstik, Paulien Klap, Imelda J. M. de Groot

**Affiliations:** 1 Department of Rehabilitation, Radboud University Medical Center, Donders Centre for Neuroscience, Reinier Postlaan 4, 6525 GC Nijmegen, The Netherlands; 2 Yumen Bionics, Amsterdam, The Netherlands

**Keywords:** Duchenne muscular dystrophy, Spinal muscular atrophy, Upper limb, Dynamic arm support

## Abstract

**Background:**

Neuromuscular disorders (NMD) commonly affect the upper extremity. Due to muscle weakness, performance of daily activities becomes increasingly difficult, which leads to reduced independence and quality of life. In order to support the performance of upper extremity tasks, dynamic arm supports may be used. The Yumen Arm is a novel dynamic arm support specially developed for people with NMD. The aim of this study is to evaluate the feasibility and effectiveness of the Yumen Arm in persons with Duchenne Muscular Dystrophy (DMD) and persons with Spinal Muscular Atrophy (SMA).

**Methods:**

Three persons with DMD and three persons with SMA participated in this study. All participants conducted a set of measures with and without the Yumen Arm. Outcome measures were: active range of motion of the arm and trunk (i.e. Reachable Workspace, Functional Workspace, and trunk movement), fatigue (OMNI-RPE), Performance of Upper Limb (PUL) scale and some additional activities of daily living. User experiences were collected using a questionnaire.

**Results:**

The Yumen Arm could be used by all participants. Results showed a median increase in active range of motion (4% relative surface area), and a median increase of function ability (>11% PUL score) when using the Yumen Arm. In addition, three out of four (data from 2 participants was missing) participants indicated that activity performance was less fatiguing when using the Yumen Arm. Four out of five (data from 1 participant was missing) participants indicated that they would like to use the Yumen Arm in their daily lives.

**Conclusion:**

This study is one of the first studies describing a range of objective measures to examine the effectiveness of a dynamic arm support. Based on these measurements we can conclude that the Yumen Arm effectively improves arm function in NMD patients, however the effectiveness varies a lot between individual subjects. We provided detailed recommendations for the improvement of the Yumen Arm, and possible also for the development of other dynamic arm supports. This study showed a lot of variability between individual subjects, which emphasizes the importance of tuning dynamic arm supports based on individual user characteristics, such as scoliosis, functional capacity and muscle strength.

**Supplementary Information:**

The online version contains supplementary material available at 10.1186/s12984-021-00868-6.

## Background

Worldwide there are over 600 different neuromuscular disorders (NMD) with an estimated total prevalence of>160/100.000 people [1]. In a large part of these disorders the upper extremity is affected [2]. Due to upper extremity muscle weakness, many patients with NMD have difficulties performing daily activities with the arms and hands. Upper extremity limitations are severely disabling and have great impact on independence and quality of life, especially as many NMD patients are wheelchair users [3]. Consequently, there is a need for treatment options that can improve upper extremity function in NMD patients. As most NMD do not have a cure yet, and medication can only delay upper extremity limitations by several years, improvement of upper extremity function can mainly be achieved using assistive technology, such as dynamic arm supports.

Over the past decades several (semi)passive dynamic arm supports, such as the Mobile Arm support (MAS) and Wilmington Robotic EXoskeleton (WREX), by JAECO Orthodedic), the Armon Ayura (Microgravity Products), the TOP/HELP and Darwing (Focal Meditech), and many more have been developed and tested [4,6]. Despite the fact that most of the dynamic arm supports that were studied improved the ability to perform activities of daily living, the use of dynamic arm supports in the home situation was reported low [4]. Although more research on this topic should be performed, several factors that could contribute to the low home use of dynamic arm supports are already identified [7, 8]. These factors can be divided in: personal factors, aesthetics, environmental factors, costs and additional factors. Personal factors are for example: user satisfaction (both with regard to the device and the services), inability to use compensatory mechanisms, difficulties in use, and clinical deterioration [8]. Aesthetics of the arm support are important for acceptance of the device. The functionality gained with the dynamic arm supports should outweigh its expense and appearance, especially for children and adolescents with NMD [9]. Environmental factors are for example: customer service (i.e. problem solving related to the device) and use of a manual wheelchair. In unpublished data of our own research group, manual wheelchair users stated that existing arm supports did not have the required range of motion to operate the wheelchair. Finally, most arm supports only provide unilateral support and restrict trunk movement, which limits the range of motion of the arms [10]. Many daily activities require trunk movement to reach for objects, or require the use of both arms or hands (bimanual tasks). The inability to support these tasks also leads to low home use.

In order to overcome some of the factors for non-use of arm supports and to optimize the effectiveness of arm supports for NMD patients, Yumen Bionics (Amsterdam, the Netherlands) developed a novel dynamic arm support; the Yumen Arm. The design of this arm support was based on a previous prototype, the passive A-Gear, which was developed for and tested with Duchenne Muscular Dystrophy (DMD) patients by our research group [11]. This previous study involved 3 boys with DMD and demonstrated that the A-gear prototype enabled them, for example, to independently drink a glass of water, raise and wave their arms, and move the wheels of their wheelchair. These tasks where not possible without the device or only for a very limited time [11]. Since the development of the passive A-Gear many improvements have been made in the design, such as an added free trunk movement structure, a slimmer spring configuration and the exoskeleton is adjustable to all sizes. The Yumen Arm has some unique features in comparison to some of the commercially available arm supports, such as (1) the Yumen Arm always supports both arms, which could increase the performance of bimanual tasks, (2) the Yumen Arm has a large range of motion, which allows users to still use their manual wheelchair, (3) the Yumen Arm has a soft/fabric lower arm cup, which reduces interference of the device with the environment, for example during handwriting, (4) the joints of the Yumen Arm are aligned with the user, which allows for natural movements, and (5) the Yumen Arm is scalable and placed close to the body, which reduces conspicuousness and improves aesthetics.

The aim of this study is to evaluate the feasibility and effectiveness of the Yumen Arm in persons with Duchenne Muscular Dystrophy (DMD) and persons with Spinal Muscular Atrophy (SMA). We hypothesize that the Yumen Arm will improve upper extremity function in persons with DMD and SMA and that the Yumen Arm will reduce fatiguability. We choose to study effectiveness of the Yumen Arm in both DMD and SMA patients as they have relatively similar upper limb function profile [12].

## Methods

### Design Yumen Arm

**Fig. 1:**
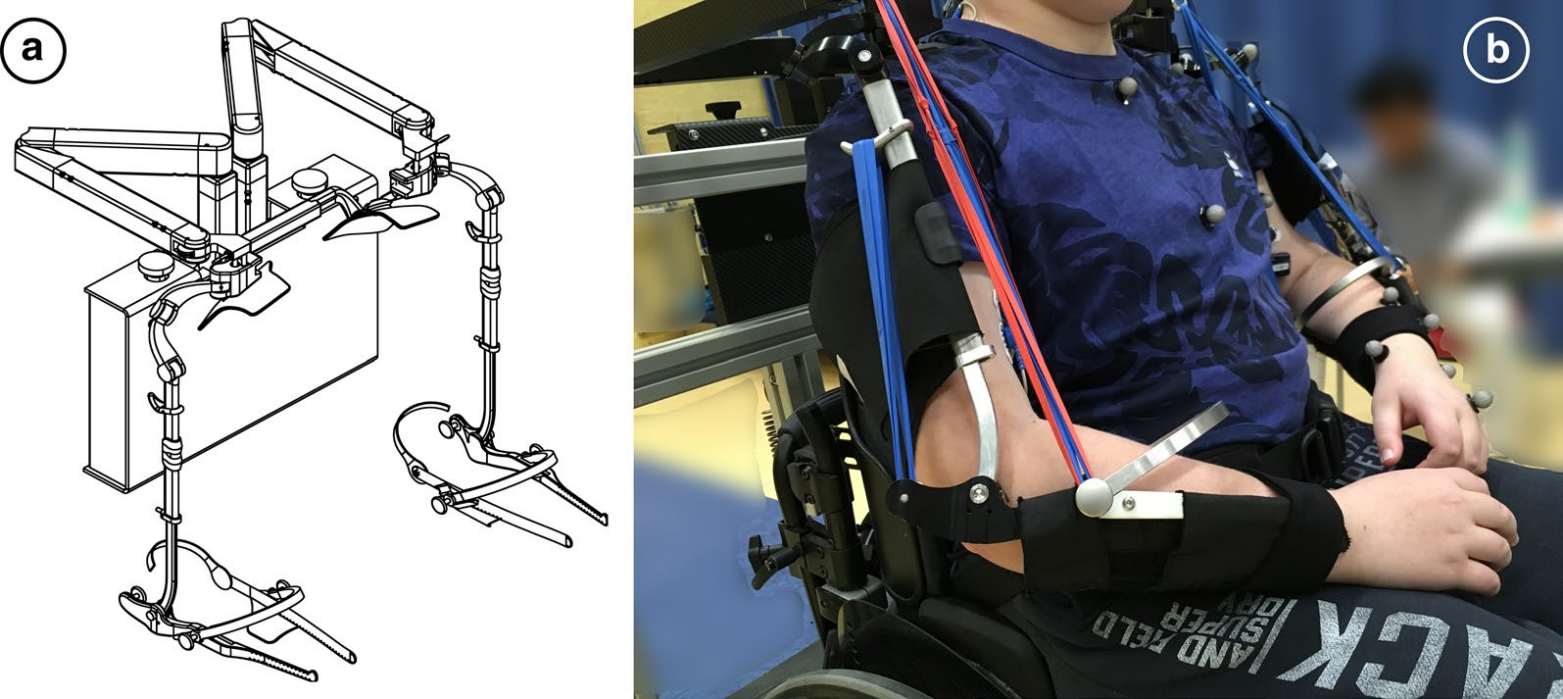
Yumen arm. **a** Design sketch without elastic bands, **b** Prototype fitted on one of the participants

The Yumen Arm is a statically balanced passive dynamic support developed by the startup company Yumen Bionics (Fig.1). Yumen Bionics derives its name from the words yume (Japanese for dreams) and human. The company was founded to translate the results of academic projects to products that can be brought to the market. The goal of Yumen Bionics is to help people with muscle weakness pursue their dream of moving independently again by offering assistive technology such as the Yumen Arm.

The mechanical design of the Yumen Arm is based on the prototype that has been developed as part of the A-gear project within the Flextension initiative [11]. This design uses strategically placed springs to maintain a static balance, a concept first described by Lin et al. [13]. This design ensures a force equilibrium in every possible position in the complete range of motion of the structure. The springs used in the Yumen Arm are bands of polyisoprene rubber with a linear range of 100% to 220% of the bands length. Rubber bands were chosen because of the extremely high strain energy density compared to metal torsion springs (16J/cm^3^ versus 0,3J/cm^3^ in metal torsion springs). The strain energy requirements of the exoskeleton requisites the use of rubber, as metal torsion springs would require more volume than there is available between the shoulder and the lower arm. Per participants a set of rubber bands is selected to realize around 70% compensation of their estimated arm weight. This percentage was chosen based on previous qualitative user research. Participants indicated that support of more than 70% of the arm weight felt less natural and less intuitive, while compensation of less than 70% of the estimated arm weight required to much effort. The rubber bands were selected for every participant individually and we tested the spring configuration during a fitting session. In this way, the weight of the arm that the participants have to lift actively is reduced significantly, thereby allowing more muscle force to be used for movement of the arm in order to carry out daily tasks, and/or doing those tasks for a longer time. The Yumen Arm is fixed to the wheelchair using a spring balanced mechanism that balances the lifted weight of the patients arm plus the weight of the exoskeleton arms itself. This allows the patient to use the exoskeleton without extra weight on their shoulders. In addition, the structure remains close to the body and allows free movement of the trunk when seated in the wheelchair. The Yumen Arm can be adjusted to the dimensions of individual participants by adjusting the length of the upper arm part, the width of the lower arm arch, and the width of the connector at the back of the participants. The primary target population of the Yumen Arm are people with neuromuscular disorders and other flaccid paresis, that use a wheelchair.

### Population

In total 3 boys with DMD and 3 persons with SMA (2 female, 1 male) participated in the study. Participants were included if they had a DNA established diagnosis of DMD or SMA, and if they had a score of 24 on the Brooke upper extremity rating scale [14]. Participants were excluded if they were younger than 7years of age, or if they had other disabling diseases that affected their upper extremity. Participants were recruited by advertisement through patient organizations Spierziekten Nederland, Prinses Beatrix Spierfonds and Duchenne Parent Project. This study was approved by the medical ethical committee ArnhemNijmegen, the Netherlands (Registration Number 20162824, NL nr.: NL58988.091.16). Informed consent was obtained before the start of the study from all participants and from their parents when the participants were under 16years of age.

Table 1 shows the characteristics of the participants in this study. Three persons with DMD and three persons with SMA were included. Brooke upper extremity functional rating scale varied between 2 (can raise arms above head only by flexing the elbow or using accessory muscles) to 4 (can raise hands to mouth, but cannot raise an 8-oz glass of water to the mouth). Next to the large differences in functional status, the study population also showed large variability in age, height and weight. Furthermore, participants had different experience levels with regard to the use of dynamic arm supports. For some participants it was their first experience with a dynamic arm support, while other participants already used a commercially available arm support in their daily lives.

**Table 1:** participant characteristics

	Subject 1	Subject 2	Subject 3	Subject 4	Subject 5	Subject 6
Diagnosis	DMD	DMD	SMA	DMD	SMA	SMA
Gender	Male	Male	Female	Male	Female	Male
Age (year)	14	18	41	11	11	28
Handedness	Right	Left	Right	Right	Right	Right
Scoliosis	No	Mild	Severe (had surgery)	No	Severe (had surgery)	Severe (had surgery)
Brooke scale	3	4	4	2	4	3
Weight (kg)	72	46	66	63	29	110
Height (m)	1.65	1.56	1.57	1.65	1.33	1.92
Wheelchair type	Manual with power assist	Manual with power assist	Electric	Manual with power assist	Electric	Electric
Fit of Yumen Arm	Good	Moderate	Bad	Good	Bad	Moderate

### Procedures

When participants agreed to participate, the researcher and technicians from Yumen Bionics visited the participants at their homes to take anthropometric dimensions of the arm and trunk and to do a first fitting of the Yumen Arm. After this visit adjustments were made to the prototype in order to match the dimensions of the Yumen Arm to the dimensions of the individual participants. During a revisit, the dimensions were checked and the correct amount of supports for balancing the arm against gravity was set by adjusting the number of elastic bands. After the revisit additional changes were made to the Yumen Arm if necessary. Finally, participants visited the movement laboratory of the Radboudumc (Nijmegen, the Netherlands) for the evaluation of the Yumen Arm in a standardized environment. During this evaluation all participants performed a series of measurements, first without the Yumen Arm and then with the Yumen Arm. In between measurements there was a break of about 1h to give the participants the opportunity to rest and minimize the effects of fatigue.

### Measurements/outcome measures

For the evaluation of the Yumen Arm we measured participant characteristic, such as age, disorder, SMA-type, occurrence of scoliosis, Brooke Upper Extremity rating scale [14], weight, height and wheelchair type. We also report the fit of the Yumen Arm as estimated by the researchers. Quality of fit was scored as good, moderate or bad, based on the relation between segment lengths and joint alignment of the Yumen Arm relative to the participants anatomy. In addition, we used outcome measures on the International Classification of Functioning, disability and health (ICF) level of body functions and structures and on ICF activity level [15]. All outcome measures were aimed to investigate the capacity of the user with and without using the Yumen Arm. Outcome measures on the ICF level of body functions and structures were active range of motion of the arm and trunk, and fatigue. Outcome measures on the ICF activity level were the Performance of Upper Limb (PUL) scale and some additional activities of daily living that were not captured within the PUL. All tasks were performed once per condition (with and without arm support). All participants were measured in their own wheelchair with back support.

#### Fatigue

Fatigue was examined using the OMNI Rating of Perceived Exertion (OMNI-RPE) scale, an 11-point scale, where 0 indicates not tired at all and 10 indicates very, very tired [16]. Participants were asked to score their level of experienced fatigue before and after the measurements with and without the Yumen Arm. We specifically asked participants to indicate the muscle fatigue experienced in the arms.

#### Range of motion

In order to gain insight in the range of motion of the arms and trunk, three different tasks were used. First, we measured reachable workspace of the hand relative to the shoulder using the reachable workspace analysis [17]. Secondly, we measured the functional workspace (ability to reach close to the body), which was an option of reachable workspace software developed by Kurillo and Han et al. [18]. Finally, we measured compensatory trunk movements during the performance of daily activities.

#### Reachable workspace

Reachable workspace analysis is an outcome measure closely related to shoulder range of motion and it is quite commonly used in neuromuscular disorders [17, 19]. In contrast to using the Microsoft Kinect, which is normally used for the reachable workspace analysis, we used a 10-camera VICON motion analysis system (Oxford Metrics, Oxford, UK) to capture 3D positions of the upper extremity joints with a sample frequency of 100Hz. Twenty-four external reflecting markers ( 14mm) were placed according to the Upper Limb Model product guide (Revision 1.0 July 2007). Reachable workspace (i.e. relative surface area representing the portion of the unit hemisphere that is covered by the hand movement) was calculated by using the joint locations from the VICON system as input for the reachable workspace software [18]. Next to the full relative surface area (RSA), the RSAs per quadrant (upper medial and lateral, lower medial and lateral) were calculated. The full RSA has a maximal value of 1, which is scored if the hand is able to reach all parts of the circle around the shoulder at 1 arm length. In addition, the absolute total and quadrant reachable workspace surface envelope areas (m^2^) (ASA) were calculated. Reachable workspace analysis was performed with both the left and right arm.

#### Functional workspace

The functional workspace analysis was performed with both the right and left hand and consisted of 7 movements in which the participants were asked to touch the following close to body locations: belly button, back pocket, ipsilateral shoulder, contralateral shoulder, mouth, top of the head and back head [20]. As there was no standardized and automated method for analyzing the functional workspace at the time of this study, we analyzed the results manually based on video recordings (videos of the frontal and sagittal plane were recorded). For each target a score of 03 was awarded by the examiner: 0=the participants was not able to perform the task, 1=the participant was able to reach to 049% of the target position, 2=the participant was able to reach to 5099% of the target position, and 3=the participant was able to fully reach the target position. Next to the score for each target separately a sum score for all targets was calculated.

#### Compensatory trunk movement

In order to gain insight in the compensatory mechanisms used with and without the Yumen Arm, we measured the amount of trunk movement during the performance of three daily tasks, i.e. drink, move a 100g weight, and use the keyboard of a computer. Increased trunk movement during task performance is a commonly used compensatory strategy [11, 21]. We measured trunk movement based on the normalized (for task duration) 3D movement (mm) of a reflective marker placed on the jugular notch during task performance.

#### Functional ability

Functional ability was examined using the high level-shoulder and mid-level-elbow dimensions of the performance of upper limb (PUL) scale [22]. PUL items were performed with both hands separately in case of unilateral tasks, for bimanual tasks both hands were used simultaneous. In addition to the PUL items we examined 6 extra daily activities (i.e. using the keyboard of a computer, eating with knife and fork, shaking hands (left and right), writing, flip through pages of a book and use a manual wheelchair (if applicable)). These activities were in our opinion not captured in items of the PUL, and they were indicated as important and impaired daily activities by DMD patients in previous research [23]. PUL items were scored based on the instruction manual (PUL for DMD 2.0 Clinical Evaluator Manual) and the additional items were awarded a score from 0 to 2 (0=not able, 1=able using compensatory movements, 2=able without compensatory movements). Scores for the individual items and sum scores were calculated.

#### User experiences

After the measurements were finished, all participants were asked to fill out a questionnaire in order to gain insight in their user experiences with the Yumen Arm. The topics of the questionnaire were related to: range of motion, amount of support, stability, safety (pressure points, risks of pinching the skin, risk of involuntary movements and risk of the arm falling out of the device), comfort, fatigue, aesthetics, donning and doffing, cleaning, compatibility with other devices and interest in using the device in daily life.

### Statistical analysis

This study is setup as a case series, meaning that observations are made on a series of individuals, all receiving the same intervention [24]. We compared the results with and without using the Yumen Arm separately for each participant by using descriptive data. Effectiveness of the Yumen Arm is measured by comparing the change with and without use of the Yumen Arm for all outcome measures. No group statistics were performed.

## Results

Table 2 shows the results of fatigue. Median fatigue scores increased with 2.0 points on the OMNI-RPE scale while participants were performing the measurements without the arm support. When using the Yumen Arm the median fatigue level increased with 1.5 points on the OMNI-RPE scale while performing the measurements. There is a large variability between the experienced fatigue of participants, as some subjects experienced hardly any fatigue (subjects 1 and 2) and some subjects experienced relatively high levels of fatigue (subjects 36).

**Table 2:** Fatigue based on OMNI-RPE scores

Subject	Without Yumen Arm			With Yumen Arm		
	Pre test	Post test	Difference postpre test	Pre test	Post test	Difference postpre test
Subject 1	0	2	2	1	2	1
Subject 2	0	1	1	2.5	3	0.5
Subject 3	3.5	5.5	2	4	5	1
Subject 4	3.5	5.5	2	4.5	6.5	2
Subject 5	3.8	7.2	3.4	4.7	7.3	2.6
Subject 6	2.3	4.7	2.4	2.8	5.3	2.5
Median (range)	2.9 (0; 3.8)	5.1 (1; 7.2)	2.0 (1; 3.4)	3.4 (2; 7.3)	5.2 (2; 7.3)	1.5 (0.5; 2.6)

**Fig. 2:**
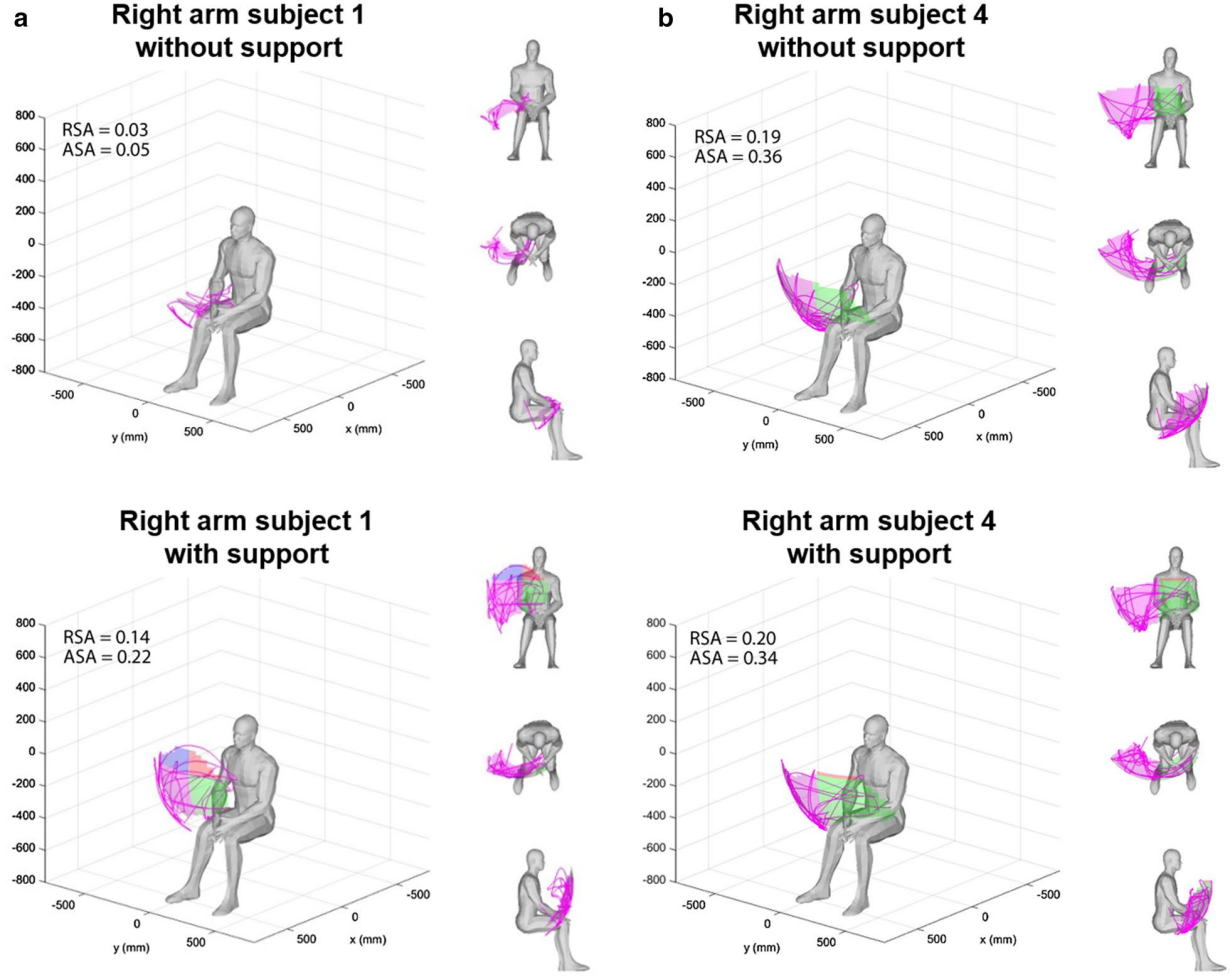
Reachable workspace with and without Yumen Arm. **a** example of subject 1, where the reachable workspace improves due to use of the Yumen Arm. **b** example of subject 4, where the reachable workspace remains unchanged when using the Yumen Arm

Table 3 shows the results of the reachable workspace analysis. The RSA of the right arm improves in 4 out of 6 participants, and de RSA of the left arm improves in 5 out of 6 participants when using the Yumen Arm. The ASA improves in 2 out of 6 participants for the right arm and in 4 out of 6 participants for the left arm. There is a large variability in the change of reachable workspace between participants. Figure2a shows an example of the reachable workspace of subject 1, who clearly improves when using the Yumen Arm. Figure2b shows an example of the reachable workspace of subject 4, who does not improve using the Yumen Arm. When looking at the changes per quadrant, the largest improvement in reachable workspace due to use of the Yumen Arm can be seen in quadrant 2, the lower medial quadrant (indicated by the green area in Fig.1). Complete results of the reachable workspace analysis including changes in each quadrant are available in Additional file 1: Appendix A1 and A2.

**Table 3:** Reachable workspace

	Right arm			Left arm		
	Without support	With support	Difference with-without support	Without support	With support	Difference with-without support
Relative surface area						
Subject 1	0.03	0.14	0.11	0.04	0.12	0.08
Subject 2	0.20	0.16	0.04	0.17	0.17	0.00
Subject 3	0.06	0.15	0.09	0.07	0.12	0.05
Subject 4	0.19	0.20	0.01	0.10	0.23	0.13
Subject 5	0.08	0.05	0.02	0.04	0.05	0.01
Subject 6	0.16	0.19	0.03	0.14	0.21	0.07
Median (range)	0.12 (0.09;0.37)	0.16 (0.05;0.20)	0.02 (0.04;0.11)	0.09 (0.04;0.17)	0.15 (0.05;0.23)	0.06 (0.00;0.13)
Absolute surface area (m^2^)						
Subject 1	0.05	0.22	0.17	0.06	0.17	0.11
Subject 2	0.33	0.25	0.07	0.26	0.20	0.05
Subject 3	0.11	0.26	0.15	0.12	0.15	0.03
Subject 4	0.36	0.34	0.02	0.18	0.31	0.13
Subject 5	0.09	0.07	0.02	0.04	0.04	0.00
Subject 6	0.37	0.36	0.00	0.28	0.37	0.08
Median (range)	0.22 (0.09;0.37)	0.26 (0.07;0.36)	0.01 (0.07;0.17)	0.15 (0.04;0.28)	0.19 (0.04;0.37)	0.06 (0.00;0.13)

Table 4 shows the results of the functional workspace analysis. The participants score as percentage of the maximal possible score is displayed. For the right arm, the ability to reach close to the body increased in 2 participants, did not change in 3 participants and decreased in 1 participant. For the left arm, the ability to reach close to the body increased in 4 participants and did not change in 2 participants. Looking at the scores of individual targets, we see that the ability to touch the top of the head and touch the ipsilateral shoulder improves for some participants, while the ability to touch the back pocket decreases when using the Yumen Arm. The ability to touch the mouth improves in some participants, but decreases in other participants when using the Yumen Arm. Results for individual targets of the functional workspace analysis can be found in Additional file 2: Appendix B.

**Table 4:** Functional workspace

	Right arm			Left arm		
	Without support	With support	Difference with-without support	Without support	With support	Difference with-without support
Percentage of maximal possible score (%)						
Subject 1	61.9	61.9	0.0	61.9	66.7	4.8
Subject 2	61.9	71.4	9.5	61.9	66.7	4.8
Subject 3	61.9	71.4	9.5	61.9	66.7	4.8
Subject 4	66.7	57.1	9.5	38.9	61.9	28.6
Subject 5	66.7	66.7	0.0	66.7	66.7	0.0
Subject 6	66.7	66.7	0.0	66.7	66.7	0.0
Median (range)	64.3 (61.9;66.7)	66.7 (57.1;71.4)	0.0 (9.5;9.5)	61.9 (38.9;66.7)	66.7 (61.9;66.7)	4.8 (0.0;28.6)

**Fig. 3:**
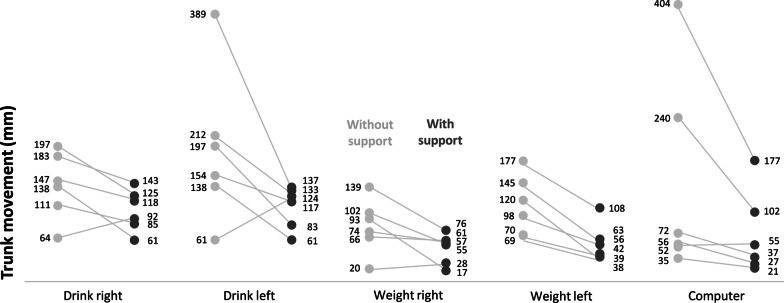
compensatory movement of the trunk (mm) during task performance with and without support. Trunk movement is defined as the absolute distance covered (mm) by the marker on the jugular notch during the following activities. Drink right: bring hand holding a cup with 200g to the mouth with the right arm; Drink left: bring hand holding a cup with 200g to the mouth with the left arm; Weight right: move a 100g weight across the table from the shoulder line to the center of the body with the right arm; Weight left: move a 100g weight across the table from the shoulder line to the center of the body with the left arm; Computer: use both hands on the keyboard of the computer to write a word

Figure3 shows the trunk movement (i.e. displacement of a marker on the sternum) that was made during the performance of 5 tasks (drinking with the left and right hand, moving a 100g weight across the table with the left and right hand, and the keyboard of a computer). In general, participants use more trunk movement while performing the tasks without support, compared to with support.

Both the results of the PUL and the additional daily activities are displayed in Table 5. Results of the PUL are expressed as the percentage of the maximal possible score for the high level-shoulder dimension, mid-level-elbow dimension and the score of the shoulder and elbow dimension together. Also, the total sum score of the additional activities are expressed as percentage of the maximal possible score. In total, we saw a median improvement of 14.5% for the right arm (equal to 4.5 points improvement) and a median improvement of 6.5% for the left arm (equal to 2.0 points improvement) when subjects where using the Yumen Arm to perform items of the PUL. For the additional activities we saw median improvements of 15.0% in both arms. None of the participant showed a decline in function when using the Yumen arm. Here also, there is a lot of variability between participants, as some participants show improvements up to 20% on PUL items and even 50% on the additional activities, while others did not show any functional improvement. When looking at individual items of the PUL, we saw that scores of abduction and flexion of the arm to shoulder height and moving a weight across the table increased most when participants use the Yumen Arm. The additional activities that showed largest improvement when using the Yumen Arm are shaking hands and turning pages of a book. Scores for individual items of the PUL can be found in Additional file 3: Appendix C1 and scores of individual added activities can be found in Additional file 3: Appendix C2.

**Table 5:** Functional ability as percent of maximal possible score (%)

	Right arm			Left arm		
	Without support	With support	Difference with-without support	Without support	With support	Difference with-without support
PUL shoulder dimension						
Subject 1	0.0	8.3	8.3	0.0	8.3	8.3
Subject 2	8.3	25.0	16.7	8.3	16.7	8.3
Subject 3	0.0	0.0	0.0	0.0	0.0	0.0
Subject 4	8.3	16.7	8.3	0.0	16.7	16.7
Subject 5	0.0	0.0	0.0	0.0	0.0	0.0
Subject 6	0.0	16.7	16.7	0.0	8.3	8.3
Median (range)	0.0 (0.0;8.3)	12.5 (0.0;25.0)	8.3 (0.0;16.7)	0.0 (0.0;8.3)	8.3 (0.0;16.7)	8.3 (0.0;16.7)
PUL elbow dimension						
Subject 1	36.8	57.9	21.1	31.6	57.9	26.3
Subject 2	57.9	68.4	10.5	57.9	57.9	0.0
Subject 3	57.9	57.9	0.0	42.1	47.4	5.3
Subject 4	52.6	73.7	21.1	47.4	68.4	21.1
Subject 5	5.3	15.8	10.5	15.8	15.8	0.0
Subject 6	63.2	78.9	15.8	63.2	73.7	10.5
Median (range)	55.3 (5.3;63.2)	63.2 (15.8;78.9)	13.2 (0.0;21.1)	44.7 (15.8;63.2)	57.9 (18.5;73.7)	7.9 (0.0;26.3)
PUL shoulder and elbow dimension						
Subject 1	22.6	38.7	16.1	19.4	38.7	19.4
Subject 2	38.7	51.6	12.9	38.7	41.9	3.2
Subject 3	35.5	35.5	0.0	25.8	29.0	3.2
Subject 4	35.5	51.6	16.1	29.0	48.4	19.4
Subject 5	3.2	9.7	6.5	9.7	9.7	0.0
Subject 6	38.7	54.8	16.1	38.7	48.4	9.7
Median (range)	35.5 (3.2;38.7)	45.2 (9.7;54.8)	14.5 (0.0;16.1)	27.4 (9.7;38.7)	40.3 (9.7;48.4)	6.5 (0.0;19.4)
Additional daily activities						
Subject 1	50.0	100.0	50.0	50.0	91.7	41.7
Subject 2	83.3	91.7	8.3	83.3	83.3	0.0
Subject 3	60.0	80.0	20.0	60.0	80.0	20.0
Subject 4	75.0	100.0	25.0	66.7	100.0	33.3
Subject 5	40.0	40.0	0.0	40.0	40.0	0.0
Subject 6	90.0	100.0	10.0	90.0	100.0	10.0
Median (range)	67.5 (40.0;90.0)	95.8 (40.0;100.0)	15.0 (0.0;50.0)	63.3 (40.0;90.0)	87.5 (40.0;100.0)	15.0 (0.0;41.7)

User experiences were examined using a questionnaire. The results from this questionnaire showed that participants experienced movements directing upward, sideward and forward as easier, because the Yumen Arms elastic bands helped in this direction. Movements directing downward and backward were experienced as more difficult when using the Yumen Arm, because the participants had to exert force in these directions as gravity was compensated and the arm had to be brought down actively. With regard to safety, most participants indicated that there were no pressure points, nor pinching of the skin, although one participant pointed out that the Yumen Arm pressed against the shoulder and another participant felt the skin was pinched between the Yumen Arm in the table near the elbow (Subject 3). In addition, the Yumen Arm did not collide with the wheelchair or table, but it sometimes collided with other parts of the body, for example the trunk or shoulder. Two participants indicated that the Yumen Arm sometimes moved to a certain position, while this was not an intended movement. None of the participants thought that the arm could fall out of the support. With regard to comfort most participants indicated that the Yumen Arm was comfortable and fitted well. The surface of the arm cup felt soft, although for some participant the hard edges of the arm cup near the elbow were a little uncomfortable. With regard to the aesthetics of the arm support participants had varying opinions, but in general they all stated that the aesthetics of the Yumen Arm can still be improved. The arm cup was liked by most participants, but the elastic bands were disliked. The metal structure was best appreciated when colored black instead of metal. Donning and doffing of the arm support was fairly fast and could most of the time be done within three minutes. Setting up the arm support for testing and finding the correct balance (one-time only setup), however took much longer (up to 45min). With regard to compatibility participants indicated that it would be hard to use the Yumen Arm in combination with a patient hoist, but that the Yumen Arm would be compatible with a PEG probe or a tracheostoma. Finally, 75%, i.e. 3 out of 4 participants (data from 2 participants was missing) of the participants indicated that moving the arm when using the Yumen Arm required less energy and therefore was less fatiguing. Four out of five participants, i.e. 80% (data from 1 participant was missing) indicated that they would be willing to use the Yumen Arm at home during daily life.

## Discussion

The aim of this study was to evaluate the feasibility and effectiveness of the Yumen Arm in people with DMD and SMA. With regard to feasibility all participants were able to fit the Yumen Arm after two fitting sessions, which was also indicated in the questionnaire, where 5 out of 6 participants stated that the Yumen Arm fitted well. Yet, the quality of fit as scored by the researchers differed a lot between de subjects (Table 1). The Yumen Arm was originally developed for boys with DMD and during the fitting process we indeed saw that the prototype fitted better in boys with DMD compared to the SMA patients. Important factors for the prototype not fitting as well in SMA patients were: scoliosis, gender and variability in body dimensions. In contrast to the DMD patients, all SMA patients suffered from severe scoliosis. As a result, SMA patients had difficulties with sitting upright and their shoulder height was not at the same level for the left and right shoulder. This asymmetry led to a worse fit of the Yumen Arm. Gender was important during the fitting process, as it appeared that the chest strap did not fit well in the adult female participant. Finally, the large variability in body dimensions of the SMA patients (height varied from 133 to 192cm and weight varied from 29 to 110kg) led to difficulties with the correctly fitting the Yumen Arm. The DMD patients included showed much more homogeneity in terms of age, gender and body dimensions and therefore the Yumen Arm was easier to fit in this population. Difficulties with fitting were mainly present in subject 3 and subject 5, the female SMA patients, who also showed the lowest functional benefit. In order to ensure feasibility of the Yumen Arm in SMA patients, future design adaptations have to be made to meet the specific needs of SMA patients, such as removal of the chest strap, building in adjustment options for patients with asymmetric shoulder height and optimize fitting with regard to joint alignment.

To examine the effectiveness of the Yumen Arm, we evaluated fatigue, range of motion, functional ability and user satisfaction. Subjectively, participants indicated that less energy was required to move the arm while using the Yumen Arm. In addition, the OMNI-RPE scores did increase less while performing the measurement with the Yumen Arm compared to the measurement without the Yumen Arm. Hereby it should be noted that all pre-test fatigue levels were higher before the test with the Yumen Arm, which is probably due to the fact that the break between the measurements was not sufficient to fully recover from the measurement without the Yumen Arm. Future studies should ensure that fatigue at the start of a measurement is not different between conditions, for example by randomizing the order in which different conditions are examined. In addition, future studies should focus on objective evaluation of fatigue levels, for example by looking at the ability to perform tasks repeatedly or by looking at muscle activity changes during fatiguing tasks, as dynamic arm supports could play an important role in reducing daily life fatigue.

Regarding range of motion, we saw varying results. Reachable workspace did increase in most of the participants (4 out of 6) when using the Yumen Arm, but with a median RSAs of 0.16 (left and right arm combined) when using the Yumen Arm, participants are only able to reach a small percentage of their total workspace. In addition, the ability to reach targets close to the body (functional workspace) shows a median increase of only 2.4 percent (left and right arm combined). Nevertheless, we saw some promising increases in range of motion of individual participants. Reachable workspace more than doubled in two participants and functional workspace improved up to 28 percent for one participant. The varying results with regard to range of motion may partly be explained by the relative high levels of fatigue during testing and by the quality of fit, as participants where the Yumen arm fitted best showed the highest improvement in range of motion. Although, it is evident that improvements should be made with regard to range of motion, the Yumen Arm might still be beneficial to participants where the range of motion did not increase, for example by assisting in functional activities and by reducing fatigue. Optimally, the Yumen Arm should give patients the ability to operate within a functional workspace that allows them to perform ADL. With regard to the inability to improve range of motion in some participants, we expect that muscle strength of these participants was insufficient to lift the 30% of the arm weight that remains after balancing the arm, and to overcome friction within the Yumen Arm, that occurs as a result of misalignment with the human joints and balancing error due to nonlinearity and hysteresis in the elastic bands. As a result, participants were still not able to lift the arm against gravity over the full range of motion while supported by the Yumen Arm. Future studies should try to determine in advance if users reach the necessary requirements and operating thresholds for the Yumen Arm. Operating thresholds are probably dependent on the ability to use compensatory mechanisms and the available muscle strength, which is very variable between subjects [25].

The use of trunk movements during the performance of daily activities, such as drinking, moving an object and using a computer, was reduced in all participants when performing the tasks with the Yumen Arm. Trunk movements are often used as compensatory mechanisms to allow task performance, which would not be possible without compensation [11]. A reduction of trunk movements when performing tasks with the Yumen Arm, may indicate that task performance is easier with the Yumen Arm as compensatory mechanisms are no longer necessary for task performance. Indeed, observing the participants we saw more natural movement patterns.

Concerning functional ability, PUL scores of the shoulder and elbow dimension showed a median increase of 11.3% (range 019.4%, i.e. 3.5 points) and the ability to perform the additional daily activities improved with a median of 15% (range 050%). In a natural history cohort, the PUL score of non-ambulant DMD patients on average decreases with 4.4 points (1.2 points on the shoulder dimension, 2.4 points on the elbow dimension and 0.8 points on the distal dimension) over a 2-year period [26]. These results indicate that functional improvements due to the Yumen Arm are similar to 2-years of decline resulting from natural disease progression, demonstrating the clinical relevance of the improvements. This comparison is unfortunately not possible for SMA patients as no natural history data based on the PUL are available in this population. The effect of the Yumen Arm on functional ability may improve more over time as participants use the device for a longer period of time (learning effect), or if they receive specific training on how to use the arm support. Therefore, we recommend future studies to look into the learning effect of arm supports and the effect of training with the arm support, for example with an occupational therapist.

Other studies that examined the effectiveness of (commercially available) dynamic arm supports in NMD patients also saw some difficulties regarding the ability of passive devices to lift the arm against gravity, especially regarding lifting the arm above shoulder level. Hasewage et al. saw that the supportive torque of the Exoskeletal meal assistance system III (EMAS III) was not sufficient to flex the shoulder upward, and concluded that gravity compensation alone is not enough to support the upper arm during various activities in people with SMA [27]. In addition, Haumont et al. concluded that the Wilmington Robotic EXoskeleton (WREX) was able to increase shoulder abduction up to 40 degrees, however the SMA patients were still not able to lift shoulder above 5075 degrees of flexion [28]. Kramer et al. concluded that users with small muscle force can use the Dynamic Arm Support (DAS) to make up and downward movements, however gravity compensation for users with very small muscle forces may be insufficient due to hysteresis and nonlinearity of the system [29]. Although the range of motion of the arms when using an arm support is often still limited, most studies show that passive arms supports increase the ability to perform daily activities [4, 30,33].

Regarding the design of the study, we would like to remark that this study only focused on the capacity (what someone can do in a controlled environment) and not on performance (what someone actually does in daily life). For future studies into the effectiveness of arm supports we recommend to include performance measures as well, as they do not only give insight in functional abilities, but also in the ability to effectively use an arm support in a daily life environment.

We performed this study in a small and heterogenous sample, which may limit the ability to interpret the results. The large variability between subjects and the study design (case series without group statistics) makes it hard to formulate a solid conclusion on the effectiveness of the Yumen Arm. On the other hand, the large variability between subjects and individual description of the results gave us important insights in the participant characteristics of people who can benefit from the Yumen Arm, and likely also from other dynamic arm supports. It became clear that the one size fits all principle does not apply for dynamic arm supports, and that there is a need for individual adaptable devices. There are multiple dynamic arm supports on the market and based on individual participants characteristics and preferences, different types of arm supports might suit some people better than others. Therefore, we recommend people in need of an arm support to try different dynamic arm support if they have the chance. Furthermore, we would like to note that the Yumen Arm was not designed to fully normalize arm function. Its dimensions, weight and ability to move also restrict task performance in some way. In addition, aesthetics of the Yumen Arm should be improved to overcome the negative effects of aesthetics on the home use of an arm support. All participants indicated that the aesthetics of the Yumen Arm can be improved. Especially the structure at the back of the wheelchair is quite large and does not follow the shape of the human body as well as the distal part of the Yumen Arm. Future research should focus on further optimization of the relation between the functional benefits and the restrictive factors of the Yumen arm. Finally, the Yumen Arm has some unique features compared to other dynamic arm supports, such as bimanual support, freedom to move the trunk and ability to reach the wheels of a manual wheelchair. Although, the participants indicated to be very positive about these features, we did not test the added benefits of these features. We recommend future studies to compare the effectiveness of the Yumen Arm, to other arm supports to gain insight is the possible benefits of these unique features.

## Conclusions

This study is one of the first studies describing a range of objective measures to examine the effectiveness of a dynamic arm support. Based on these measurements we can conclude that the Yumen Arm effectively improves arm function in NMD patients, especially in people with DMD. However the effectiveness varies a lot between individual subjects and the prototype Yumen Arm should be improved before it can be commercialized. We provided detailed recommendations for further improvement of the Yumen Arm, and possible also for the development of other arm exoskeletons. This study showed a lot of variability between individual subjects, which emphasizes the importance of tuning dynamic arm supports based on individual user characteristics, such as scoliosis, functional capacity and muscle strength.

## Supplementary Information

**Additional file 1: Appendix A.** Reachable workspace full result.

**Additional file 2: Appendix B.** Functional workspace score per item.

**Additional file 3: Appendix C. **Functional ability**.**

## Data Availability

The datasets used and/or analyzed during the current study are available from the corresponding author upon reasonable request.

## Abbreviations

DMD: Duchenne Muscular Dystrophy
SMA: Spinal Muscular Atrophy
NMD: Neuromuscular disorders
OMNI-RPE: OMNI Rating of Perceived Exertion
PUL: Performance of Upper Limb
ICF: International Classification for Functioning, Disability and Health
RSA: Relative surface area
ASA: Absolute surface area
PEG: Percutaneous endoscopic gastrostomy
EMAS: Exoskeletal meal assistance system
WREX: Wilmington Robotic EXoskeleton
DAS: Dynamic Arm Support
