# Supplementary material for: Feasibility and effectiveness of a novel dynamic arm support in persons with spinal muscular atrophy and duchenne muscular dystrophy
Source: J Neuroeng Rehabil. 2021 May 21;18:84. doi: 10.1186/s12984-021-00868-6 (PMC8139063; doi:10.1186/s12984-021-00868-6)
Supplement: Supplementary file 1 — Additional file 1: Appendix A. Reachable workspace full result. [file 12984_2021_868_MOESM1_ESM.pdf]

Appendix A. Reachable workspace – full result

Table A1: Relative surface area

	Right arm			Left arm		
	**Without support**	**With support**	**Difference with-without support**	**Without support**	**With support**	**Difference with-without support**
RSA Quadrant 1 – Upper medial						
Subject 1	0.00	0.01	0.01	0.00	0.00	0.00
Subject 2	0.00	0.00	0.00	0.00	0.03	0.03
Subject 3	0.00	0.00	0.00	0.00	0.00	0.00
Subject 4	0.00	0.00	0.00	0.00	0.02	0.02
Subject 5	0.00	0.00	0.00	0.00	0.00	0.00
Subject 6	0.00	0.01	0.01	0.00	0.00	0.00
RSA Quadrant 2 – Lower medial						
Subject 1	0.01	0.04	0.03	0.02	0.05	0.03
Subject 2	0.05	0.04	-0.01	0.07	0.07	0.00
Subject 3	0.00	0.04	0.04	0.01	0.03	0.03
Subject 4	0.06	0.09	0.03	0.05	0.11	0.06
Subject 5	0.01	0.00	0.00	0.00	0.00	0.00
Subject 6	0.04	0.05	0.01	0.06	0.08	0.02
RSA Quadrant 3 – Upper lateral						
Subject 1	0.00	0.02	0.02	0.00	0.01	0.01
Subject 2	0.01	0.01	0.00	0.00	0.00	0.00
Subject 3	0.00	0.00	0.00	0.00	0.00	0.00
Subject 4	0.00	0.00	0.00	0.00	0.00	0.00
Subject 5	0.00	0.00	0.00	0.00	0.02	0.02
Subject 6	0.00	0.02	0.02	0.00	0.01	0.01
RSA Quadrant 4 – Lower lateral						
Subject 1	0.03	0.07	0.05	0.02	0.06	0.04
Subject 2	0.14	0.11	-0.03	0.09	0.07	-0.02
Subject 3	0.06	0.11	0.05	0.07	0.09	0.02
Subject 4	0.13	0.11	-0.02	0.05	0.10	0.04
Subject 5	0.07	0.05	-0.02	0.04	0.03	-0.01
Subject 6	0.12	0.12	-0.01	0.08	0.12	0.04
RSA - total						
Subject 1	0.03	0.14	0.11	0.04	0.12	0.08
Subject 2	0.20	0.16	-0.04	0.17	0.17	0.00
Subject 3	0.06	0.15	0.09	0.07	0.12	0.05
Subject 4	0.19	0.20	0.01	0.10	0.23	0.13
Subject 5	0.08	0.05	-0.02	0.04	0.05	0.01
Subject 6	0.16	0.19	0.03	0.14	0.21	0.07

Table A2: Absolute surface area (m^2)

	Right arm			Left arm		
	**Without support**	**With support**	**Difference with-without support**	**Without support**	**With support**	**Difference with-without support**
RSA Quadrant 1 – Upper medial						
Subject 1	0.00	0.02	0.02	0.00	0.00	0.00
Subject 2	0.00	0.00	0.00	0.00	0.03	0.03
Subject 3	0.00	0.00	0.00	0.00	0.00	0.00
Subject 4	0.00	0.01	0.01	0.00	0.03	0.03
Subject 5	0.00	0.00	0.00	0.00	0.00	0.00
Subject 6	0.00	0.01	0.01	0.00	0.00	0.00
RSA Quadrant 2 – Lower medial						
Subject 1	0.01	0.06	0.05	0.03	0.06	0.04
Subject 2	0.08	0.06	-0.01	0.11	0.09	-0.03
Subject 3	0.01	0.08	0.07	0.01	0.04	0.03
Subject 4	0.12	0.15	0.03	0.09	0.15	0.06
Subject 5	0.01	0.00	0.00	0.00	0.00	0.00
Subject 6	0.08	0.09	0.01	0.12	0.14	0.02
RSA Quadrant 3 – Upper lateral						
Subject 1	0.00	0.03	0.03	0.00	0.01	0.01
Subject 2	0.02	0.02	0.00	0.00	0.00	0.00
Subject 3	0.00	0.00	0.00	0.00	0.00	0.00
Subject 4	0.00	0.00	0.00	0.00	0.00	0.00
Subject 5	0.00	0.00	0.00	0.00	0.01	0.01
Subject 6	0.00	0.04	0.04	0.00	0.02	0.02
RSA Quadrant 4 – Lower lateral						
Subject 1	0.04	0.11	0.08	0.03	0.09	0.06
Subject 2	0.22	0.16	-0.06	0.14	0.08	-0.06
Subject 3	0.10	0.19	0.08	0.11	0.11	0.00
Subject 4	0.24	0.18	-0.06	0.09	0.13	0.04
Subject 5	0.08	0.07	-0.02	0.04	0.03	-0.02
Subject 6	0.28	0.21	-0.07	0.17	0.21	0.04
RSA - total						
Subject 1	0.05	0.22	0.17	0.06	0.17	0.11
Subject 2	0.33	0.25	-0.07	0.26	0.20	-0.05
Subject 3	0.11	0.26	0.15	0.12	0.15	0.03
Subject 4	0.36	0.34	-0.02	0.18	0.31	0.13
Subject 5	0.09	0.07	-0.02	0.04	0.04	0.00
Subject 6	0.37	0.36	0.00	0.28	0.37	0.08
