# Supplementary material for: Feasibility and effectiveness of a novel dynamic arm support in persons with spinal muscular atrophy and duchenne muscular dystrophy
Source: J Neuroeng Rehabil. 2021 May 21;18:84. doi: 10.1186/s12984-021-00868-6 (PMC8139063; doi:10.1186/s12984-021-00868-6)
Supplement: Supplementary file 2 — Additional file 2: Appendix B. Functional workspace score per item. [file 12984_2021_868_MOESM2_ESM.pdf]

Appendix B. Functional workspace – score per item

Table B: Functional workspace per item

	Right arm			Left arm		
	**Without support**	**With support**	**Difference with-without support**	**Without support**	**With support**	**Difference with-without support**
Target – belly button						
Subject 1	3	3	0	3	3	0
Subject 2	3	3	0	3	3	0
Subject 3	3	3	0	3	3	0
Subject 4	3	3	0	3	3	0
Subject 5	3	3	0	3	3	0
Subject 6	3	3	0	3	3	0
Target – back pocket						
Subject 1	1	0	-1	1	1	0
Subject 2	2	2	0	2	1	-1
Subject 3	1	1	0	1	0	-1
Subject 4	2	1	-1	.	1	.
Subject 5	.	.	.	.	.	.
Subject 6	.	.	.	.	.	.
Target – ipsilateral shoulder						
Subject 1	1	2	1	2	2	0
Subject 2	1	2	1	1	2	1
Subject 3	3	3	0	3	3	0
Subject 4	1	1	0	1	2	1
Subject 5	2	2	0	2	2	0
Subject 6	2	2	0	2	2	0
Target – contralateral shoulder						
Subject 1	2	2	0	2	2	0
Subject 2	2	2	0	2	3	1
Subject 3	2	2	0	2	2	0
Subject 4	3	2	-1	2	2	0
Subject 5	2	2	0	2	2	0
Subject 6	2	2	0	2	2	0
Target – mouth						
Subject 1	2	2	0	2	2	0
Subject 2	2	2	0	2	2	0
Subject 3	2	3	1	2	3	1
Subject 4	2	2	0	1	2	1
Subject 5	3	2	-1	3	2	-1
Subject 6	3	2	-1	3	2	-1
Target – top of head						
Subject 1	2	2	0	1	2	1
Subject 2	2	2	0	2	2	0
Subject 3	1	2	1	1	2	1
Subject 4	2	2	0	0	2	2
Subject 5	1	2	1	1	2	1
Subject 6	1	2	1	1	2	1
Target – back of head						
Subject 1	2	2	0	2	2	0
Subject 2	1	2	1	1	1	0
Subject 3	1	1	0	1	1	0
Subject 4	1	1	0	0	1	1
Subject 5	1	1	0	1	1	0
Subject 6	1	1	0	1	1	0

. = missing variable
