# Supplementary material for: Feasibility and effectiveness of a novel dynamic arm support in persons with spinal muscular atrophy and duchenne muscular dystrophy
Source: J Neuroeng Rehabil. 2021 May 21;18:84. doi: 10.1186/s12984-021-00868-6 (PMC8139063; doi:10.1186/s12984-021-00868-6)
Supplement: Supplementary file 3 — Additional file 3: Appendix C. Functional ability. [file 12984_2021_868_MOESM3_ESM.pdf]

# Appendix C: Functional ability

**Table C1: Performance of upper limb scores per item**

| Item | Item nr. | Side | Dimension | Subject | Without support | With support | Difference with-without support |
|---|---|---|---|---|---|---|---|
| Entry item | | | | | 3 | 3 | 0 |
| Shoulder abduction arms above head | 1 | Both arms | Shoulder | Subject 1 | 0 | 0 | 0 |
| Raise both arms to shoulder height | 2 | Both arms | Shoulder | Subject 1 | 0 | 0 | 0 |
| Shoulder flexion to shoulder height (no weights) | 3 | Right | Shoulder | Subject 1 | 0 | 1 | 1 |
| Shoulder flexion to shoulder height (no weights) | 3 | Left | Shoulder | Subject 1 | 0 | 1 | 1 |
| Shoulder flexion to shoulder height with 500g | 4 | Right | Shoulder | Subject 1 | 0 | 0 | 0 |
| Shoulder flexion to shoulder height with 500g | 4 | Left | Shoulder | Subject 1 | 0 | 0 | 0 |
| Shoulder flexion above shoulder with 500g weight | 5 | Right | Shoulder | Subject 1 | 0 | 0 | 0 |
| Shoulder flexion above shoulder with 500g weight | 5 | Left | Shoulder | Subject 1 | 0 | 0 | 0 |
| Shoulder flexion above shoulder with 1 kg weight | 6 | Right | Shoulder | Subject 1 | 0 | 0 | 0 |
| Shoulder flexion above shoulder with 1 kg weight | 6 | Left | Shoulder | Subject 1 | 0 | 0 | 0 |
| Hand(s) to mouth | 7 | Right | Elbow | Subject 1 | 1 | 1 | 0 |
| Hand(s) to mouth | 7 | Left | Elbow | Subject 1 | 1 | 1 | 0 |
| Hands to table from lap | 8 | Both arms | Elbow | Subject 1 | 1 | 2 | 1 |
| Move weight on table 100g | 9 | Right | Elbow | Subject 1 | 1 | 2 | 1 |
| Move weight on table 100g | 9 | Left | Elbow | Subject 1 | 1 | 2 | 1 |
| Move weight on table 500g | 10 | Right | Elbow | Subject 1 | 1 | 2 | 1 |
| Move weight on table 500g | 10 | Left | Elbow | Subject 1 | 0 | 2 | 2 |
| Move weight on table 1kg | 11 | Right | Elbow | Subject 1 | 0 | 1 | 1 |
| Move weight on table 1kg | 11 | Left | Elbow | Subject 1 | 0 | 1 | 1 |
| Lift heavy can diagonally | 12 | Right | Elbow | Subject 1 | 0 | 0 | 0 |
| Lift heavy can diagonally | 12 | Left | Elbow | Subject 1 | 0 | 0 | 0 |
| Stack of three cans (heavy cans) | 13 | Right | Elbow | Subject 1 | 0 | 0 | 0 |
| Stack of three cans (heavy cans) | 13 | Left | Elbow | Subject 1 | 0 | 0 | 0 |
| Stack of five cans (heavy cans) | 14 | Right | Elbow | Subject 1 | 0 | 0 | 0 |
| Stack of five cans (heavy cans) | 14 | Left | Elbow | Subject 1 | 0 | 0 | 0 |
| Remove lid from container | 15 | Both arms | Elbow | Subject 1 | 1 | 1 | 0 |
| Tearing paper | 16 | Both arms | Elbow | Subject 1 | 2 | 2 | 0 |
| Entry item | | | | Subject 2 | 3 | 4 | 1 |
| Shoulder abduction arms above head | 1 | Both arms | Shoulder | Subject 2 | 0 | 0 | 0 |
| Raise both arms to shoulder height | 2 | Both arms | Shoulder | Subject 2 | 0 | 2 | 2 |
| Shoulder flexion to shoulder height (no weights) | 3 | Right | Shoulder | Subject 2 | 1 | 1 | 0 |
| Shoulder flexion to shoulder height (no weights) | 3 | Left | Shoulder | Subject 2 | 1 | 0 | -1 |
| Shoulder flexion to shoulder height with 500g | 4 | Right | Shoulder | Subject 2 | 0 | 0 | 0 |
| Shoulder flexion to shoulder height with 500g | 4 | Left | Shoulder | Subject 2 | 0 | 0 | 0 |
| Shoulder flexion above shoulder with 500g weight | 5 | Right | Shoulder | Subject 2 | 0 | 0 | 0 |
| Shoulder flexion above shoulder with 500g weight | 5 | Left | Shoulder | Subject 2 | 0 | 0 | 0 |
| Shoulder flexion above shoulder with 1 kg weight | 6 | Right | Shoulder | Subject 2 | 0 | 0 | 0 |
| Shoulder flexion above shoulder with 1 kg weight | 6 | Left | Shoulder | Subject 2 | 0 | 0 | 0 |
| Hand(s) to mouth | 7 | Right | Elbow | Subject 2 | 1 | 1 | 0 |
| Hand(s) to mouth | 7 | Left | Elbow | Subject 2 | 1 | 1 | 0 |
| Hands to table from lap | 8 | Both arms | Elbow | Subject 2 | 2 | 2 | 0 |
| Move weight on table 100g | 9 | Right | Elbow | Subject 2 | 2 | 2 | 0 |
| Move weight on table 100g | 9 | Left | Elbow | Subject 2 | 2 | 2 | 0 |
| Move weight on table 500g | 10 | Right | Elbow | Subject 2 | 1 | 2 | 1 |
| Move weight on table 500g | 10 | Left | Elbow | Subject 2 | 1 | 1 | 0 |
| Move weight on table 1kg | 11 | Right | Elbow | Subject 2 | 1 | 1 | 0 |
| Move weight on table 1kg | 11 | Left | Elbow | Subject 2 | 1 | 1 | 0 |
| Lift heavy can diagonally | 12 | Right | Elbow | Subject 2 | 1 | 1 | 0 |
| Lift heavy can diagonally | 12 | Left | Elbow | Subject 2 | 1 | 1 | 0 |
| Stack of three cans (heavy cans) | 13 | Right | Elbow | Subject 2 | 0 | 1 | 1 |
| Stack of three cans (heavy cans) | 13 | Left | Elbow | Subject 2 | 0 | 0 | 0 |

(repeat)

| Item | Item nr. | Side | Dimension | Subject | Without support | With support | Difference with-without support |
|---|---|---|---|---|---|---|---|
| Stack of five cans (heavy cans) | 14 | Right | Elbow | Subject 2 | 0 | 0 | 0 |
| Stack of five cans (heavy cans) | 14 | Left | Elbow | Subject 2 | 0 | 0 | 0 |
| Remove lid from container | 15 | Both arms | Elbow | Subject 2 | 1 | 1 | 0 |
| Tearing paper | 16 | Both arms | Elbow | Subject 2 | 2 | 2 | 0 |
| Entry item | | | | Subject 3 | 3 | 3 | 0 |
| Shoulder abduction arms above head | 1 | Both arms | Shoulder | Subject 3 | 0 | 0 | 0 |
| Raise both arms to shoulder height | 2 | Both arms | Shoulder | Subject 3 | 0 | 0 | 0 |
| Shoulder flexion to shoulder height (no weights) | 3 | Right | Shoulder | Subject 3 | 0 | 0 | 0 |
| Shoulder flexion to shoulder height (no weights) | 3 | Left | Shoulder | Subject 3 | 0 | 0 | 0 |
| Shoulder flexion to shoulder height with 500g | 4 | Right | Shoulder | Subject 3 | 0 | 0 | 0 |
| Shoulder flexion to shoulder height with 500g | 4 | Left | Shoulder | Subject 3 | 0 | 0 | 0 |
| Shoulder flexion above shoulder with 500g weight | 5 | Right | Shoulder | Subject 3 | 0 | 0 | 0 |
| Shoulder flexion above shoulder with 500g weight | 5 | Left | Shoulder | Subject 3 | 0 | 0 | 0 |
| Shoulder flexion above shoulder with 1 kg weight | 6 | Right | Shoulder | Subject 3 | 0 | 0 | 0 |
| Shoulder flexion above shoulder with 1 kg weight | 6 | Left | Shoulder | Subject 3 | 0 | 0 | 0 |
| Hand(s) to mouth | 7 | Right | Elbow | Subject 3 | 2 | 2 | 0 |
| Hand(s) to mouth | 7 | Left | Elbow | Subject 3 | 1 | 1 | 0 |
| Hands to table from lap | 8 | Both arms | Elbow | Subject 3 | 2 | 2 | 0 |
| Move weight on table 100g | 9 | Right | Elbow | Subject 3 | 2 | 2 | 0 |
| Move weight on table 100g | 9 | Left | Elbow | Subject 3 | 2 | 2 | 0 |
| Move weight on table 500g | 10 | Right | Elbow | Subject 3 | 2 | 2 | 0 |
| Move weight on table 500g | 10 | Left | Elbow | Subject 3 | 1 | 1 | 0 |
| Move weight on table 1kg | 11 | Right | Elbow | Subject 3 | 1 | 1 | 0 |
| Move weight on table 1kg | 11 | Left | Elbow | Subject 3 | 0 | 1 | 1 |
| Lift heavy can diagonally | 12 | Right | Elbow | Subject 3 | 0 | 0 | 0 |
| Lift heavy can diagonally | 12 | Left | Elbow | Subject 3 | 0 | 0 | 0 |
| Stack of three cans (heavy cans) | 13 | Right | Elbow | Subject 3 | 0 | 0 | 0 |
| Stack of three cans (heavy cans) | 13 | Left | Elbow | Subject 3 | 0 | 0 | 0 |
| Stack of five cans (heavy cans) | 14 | Right | Elbow | Subject 3 | 0 | 0 | 0 |
| Stack of five cans (heavy cans) | 14 | Left | Elbow | Subject 3 | 0 | 0 | 0 |
| Remove lid from container | 15 | Both arms | Elbow | Subject 3 | 0 | 0 | 0 |
| Tearing paper | 16 | Both arms | Elbow | Subject 3 | 2 | 2 | 0 |
| Entry item | | | | Subject 4 | 3 | 3 | 0 |
| Shoulder abduction arms above head | 1 | Both arms | Shoulder | Subject 4 | 0 | 0 | 0 |
| Raise both arms to shoulder height | 2 | Both arms | Shoulder | Subject 4 | 0 | 1 | 1 |
| Shoulder flexion to shoulder height (no weights) | 3 | Right | Shoulder | Subject 4 | 1 | 1 | 0 |
| Shoulder flexion to shoulder height (no weights) | 3 | Left | Shoulder | Subject 4 | 0 | 1 | 1 |
| Shoulder flexion to shoulder height with 500g | 4 | Right | Shoulder | Subject 4 | 0 | 0 | 0 |
| Shoulder flexion to shoulder height with 500g | 4 | Left | Shoulder | Subject 4 | 0 | 0 | 0 |
| Shoulder flexion above shoulder with 500g weight | 5 | Right | Shoulder | Subject 4 | 0 | 0 | 0 |
| Shoulder flexion above shoulder with 500g weight | 5 | Left | Shoulder | Subject 4 | 0 | 0 | 0 |
| Shoulder flexion above shoulder with 1 kg weight | 6 | Right | Shoulder | Subject 4 | 0 | 0 | 0 |
| Shoulder flexion above shoulder with 1 kg weight | 6 | Left | Shoulder | Subject 4 | 0 | 0 | 0 |
| Hand(s) to mouth | 7 | Right | Elbow | Subject 4 | 0 | 2 | 2 |
| Hand(s) to mouth | 7 | Left | Elbow | Subject 4 | 0 | 2 | 2 |
| Hands to table from lap | 8 | Both arms | Elbow | Subject 4 | 2 | 2 | 0 |
| Move weight on table 100g | 9 | Right | Elbow | Subject 4 | 2 | 2 | 0 |
| Move weight on table 100g | 9 | Left | Elbow | Subject 4 | 2 | 2 | 0 |
| Move weight on table 500g | 10 | Right | Elbow | Subject 4 | 1 | 2 | 1 |
| Move weight on table 500g | 10 | Left | Elbow | Subject 4 | 1 | 1 | 0 |
| Move weight on table 1kg | 11 | Right | Elbow | Subject 4 | 1 | 1 | 0 |
| Move weight on table 1kg | 11 | Left | Elbow | Subject 4 | 0 | 1 | 1 |
| Lift heavy can diagonally | 12 | Right | Elbow | Subject 4 | 1 | 2 | 1 |
| Lift heavy can diagonally | 12 | Left | Elbow | Subject 4 | 1 | 2 | 1 |
| Stack of three cans (heavy cans) | 13 | Right | Elbow | Subject 4 | 0 | 0 | 0 |

(repeat)

| Item | Item nr. | Side | Dimension | Subject | Without support | With support | Difference with-without support |
|---|---|---|---|---|---|---|---|
| Stack of three cans (heavy cans) | 13 | Left | Elbow | Subject 4 | 0 | 0 | 0 |
| Stack of five cans (heavy cans) | 14 | Right | Elbow | Subject 4 | 0 | 0 | 0 |
| Stack of five cans (heavy cans) | 14 | Left | Elbow | Subject 4 | 0 | 0 | 0 |
| Remove lid from container | 15 | Both arms | Elbow | Subject 4 | 1 | 1 | 0 |
| Tearing paper | 16 | Both arms | Elbow | Subject 4 | 2 | 2 | 0 |
| Entry item | | | | Subject 5 | 3 | 3 | 0 |
| Shoulder abduction arms above head | 1 | Both arms | Shoulder | Subject 5 | 0 | 0 | 0 |
| Raise both arms to shoulder height | 2 | Both arms | Shoulder | Subject 5 | 0 | 0 | 0 |
| Shoulder flexion to shoulder height (no weights) | 3 | Right | Shoulder | Subject 5 | 0 | 0 | 0 |
| Shoulder flexion to shoulder height (no weights) | 3 | Left | Shoulder | Subject 5 | 0 | 0 | 0 |
| Shoulder flexion to shoulder height with 500g | 4 | Right | Shoulder | Subject 5 | 0 | 0 | 0 |
| Shoulder flexion to shoulder height with 500g | 4 | Left | Shoulder | Subject 5 | 0 | 0 | 0 |
| Shoulder flexion above shoulder with 500g weight | 5 | Right | Shoulder | Subject 5 | 0 | 0 | 0 |
| Shoulder flexion above shoulder with 500g weight | 5 | Left | Shoulder | Subject 5 | 0 | 0 | 0 |
| Shoulder flexion above shoulder with 1 kg weight | 6 | Right | Shoulder | Subject 5 | 0 | 0 | 0 |
| Shoulder flexion above shoulder with 1 kg weight | 6 | Left | Shoulder | Subject 5 | 0 | 0 | 0 |
| Hand(s) to mouth | 7 | Right | Elbow | Subject 5 | 1 | 1 | 0 |
| Hand(s) to mouth | 7 | Left | Elbow | Subject 5 | 1 | 1 | 0 |
| Hands to table from lap | 8 | Both arms | Elbow | Subject 5 | 0 | 0 | 0 |
| Move weight on table 100g | 9 | Right | Elbow | Subject 5 | 0 | 2 | 2 |
| Move weight on table 100g | 9 | Left | Elbow | Subject 5 | 1 | 2 | 1 |
| Move weight on table 500g | 10 | Right | Elbow | Subject 5 | 0 | 0 | 0 |
| Move weight on table 500g | 10 | Left | Elbow | Subject 5 | 1 | 0 | -1 |
| Move weight on table 1kg | 11 | Right | Elbow | Subject 5 | 0 | 0 | 0 |
| Move weight on table 1kg | 11 | Left | Elbow | Subject 5 | 0 | 0 | 0 |
| Lift heavy can diagonally | 12 | Right | Elbow | Subject 5 | 0 | 0 | 0 |
| Lift heavy can diagonally | 12 | Left | Elbow | Subject 5 | 0 | 0 | 0 |
| Stack of three cans (heavy cans) | 13 | Right | Elbow | Subject 5 | 0 | 0 | 0 |
| Stack of three cans (heavy cans) | 13 | Left | Elbow | Subject 5 | 0 | 0 | 0 |
| Stack of five cans (heavy cans) | 14 | Right | Elbow | Subject 5 | 0 | 0 | 0 |
| Stack of five cans (heavy cans) | 14 | Left | Elbow | Subject 5 | 0 | 0 | 0 |
| Remove lid from container | 15 | Both arms | Elbow | Subject 5 | 0 | 0 | 0 |
| Tearing paper | 16 | Both arms | Elbow | Subject 5 | 0 | 0 | 0 |
| Entry item | | | | Subject 6 | 3 | 3 | 0 |
| Shoulder abduction arms above head | 1 | Both arms | Shoulder | Subject 6 | 0 | 0 | 0 |
| Raise both arms to shoulder height | 2 | Both arms | Shoulder | Subject 6 | 0 | 0 | 0 |
| Shoulder flexion to shoulder height (no weights) | 3 | Right | Shoulder | Subject 6 | 0 | 2 | 2 |
| Shoulder flexion to shoulder height (no weights) | 3 | Left | Shoulder | Subject 6 | 0 | 1 | 1 |
| Shoulder flexion to shoulder height with 500g | 4 | Right | Shoulder | Subject 6 | 0 | 0 | 0 |
| Shoulder flexion to shoulder height with 500g | 4 | Left | Shoulder | Subject 6 | 0 | 0 | 0 |
| Shoulder flexion above shoulder with 500g weight | 5 | Right | Shoulder | Subject 6 | 0 | 0 | 0 |
| Shoulder flexion above shoulder with 500g weight | 5 | Left | Shoulder | Subject 6 | 0 | 0 | 0 |
| Shoulder flexion above shoulder with 1 kg weight | 6 | Right | Shoulder | Subject 6 | 0 | 0 | 0 |
| Shoulder flexion above shoulder with 1 kg weight | 6 | Left | Shoulder | Subject 6 | 0 | 0 | 0 |
| Hand(s) to mouth | 7 | Right | Elbow | Subject 6 | 2 | 2 | 0 |
| Hand(s) to mouth | 7 | Left | Elbow | Subject 6 | 2 | 2 | 0 |
| Hands to table from lap | 8 | Both arms | Elbow | Subject 6 | 2 | 2 | 0 |
| Move weight on table 100g | 9 | Right | Elbow | Subject 6 | 2 | 2 | 0 |
| Move weight on table 100g | 9 | Left | Elbow | Subject 6 | 2 | 2 | 0 |
| Move weight on table 500g | 10 | Right | Elbow | Subject 6 | 2 | 2 | 0 |
| Move weight on table 500g | 10 | Left | Elbow | Subject 6 | 2 | 2 | 0 |
| Move weight on table 1kg | 11 | Right | Elbow | Subject 6 | 1 | 2 | 1 |
| Move weight on table 1kg | 11 | Left | Elbow | Subject 6 | 1 | 1 | 0 |
| Lift heavy can diagonally | 12 | Right | Elbow | Subject 6 | 1 | 2 | 1 |
| Lift heavy can diagonally | 12 | Left | Elbow | Subject 6 | 1 | 2 | 1 |

(repeat)

| Item | Item nr. | Side | Dimension | Subject | Without support | With support | Difference with-without support |
|---|---|---|---|---|---|---|---|
| Stack of three cans (heavy cans) | 13 | Right | Elbow | Subject 6 | 1 | 2 | 1 |
| Stack of three cans (heavy cans) | 13 | Left | Elbow | Subject 6 | 1 | 2 | 1 |
| Stack of five cans (heavy cans) | 14 | Right | Elbow | Subject 6 | 0 | 0 | 0 |
| Stack of five cans (heavy cans) | 14 | Left | Elbow | Subject 6 | 0 | 0 | 0 |
| Remove lid from container | 15 | Both arms | Elbow | Subject 6 | 0 | 0 | 0 |
| Tearing paper | 16 | Both arms | Elbow | Subject 6 | 1 | 1 | 0 |

**Table C1: Additional activity scores per item**

| Item | Side | Subject | Without support | With support | Difference with-without support |
|---|---|---|---|---|---|
| Use keyboard of a computer | Both arms | Subject 1 | 1 | 2 | 1 |
| Eat with knife and fork | Both arms | Subject 1 | 1 | 2 | 1 |
| Shake hands | Right | Subject 1 | 0 | 2 | 2 |
| Shake hands | Left | Subject 1 | 0 | 1 | 1 |
| Write | Both arms | Subject 1 | 2 | 2 | 0 |
| Turn pages of a book | Both arms | Subject 1 | 1 | 2 | 1 |
| Use manual wheelchair | Both arms | Subject 1 | 1 | 2 | 1 |
| Use keyboard of a computer | Both arms | Subject 2 | 2 | 2 | 0 |
| Eat with knife and fork | Both arms | Subject 2 | 1 | 1 | 0 |
| Shake hands | Right | Subject 2 | 1 | 2 | 1 |
| Shake hands | Left | Subject 2 | 1 | 1 | 0 |
| Write | Both arms | Subject 2 | 2 | 2 | 0 |
| Turn pages of a book | Both arms | Subject 2 | 2 | 2 | 0 |
| Use manual wheelchair | Both arms | Subject 2 | 2 | 2 | 0 |
| Use keyboard of a computer | Both arms | Subject 3 | 1 | 2 | 1 |
| Eat with knife and fork | Both arms | Subject 3 | 2 | 2 | 0 |
| Shake hands | Right | Subject 3 | 0 | 0 | 0 |
| Shake hands | Left | Subject 3 | 0 | 0 | 0 |
| Write | Both arms | Subject 3 | 2 | 2 | 0 |
| Turn pages of a book | Both arms | Subject 3 | 1 | 2 | 1 |
| Use manual wheelchair | Both arms | Subject 3 | n.a. | n.a. | n.a. |
| Use keyboard of a computer | Both arms | Subject 4 | 2 | 2 | 0 |
| Eat with knife and fork | Both arms | Subject 4 | 1 | 2 | 1 |
| Shake hands | Right | Subject 4 | 1 | 2 | 1 |
| Shake hands | Left | Subject 4 | 0 | 2 | 2 |
| Write | Both arms | Subject 4 | 2 | 2 | 0 |
| Turn pages of a book | Both arms | Subject 4 | 1 | 2 | 1 |
| Use manual wheelchair | Both arms | Subject 4 | 2 | 2 | 0 |
| Use keyboard of a computer | Both arms | Subject 5 | 1 | 1 | 0 |
| Eat with knife and fork | Both arms | Subject 5 | 1 | 0 | -1 |
| Shake hands | Right | Subject 5 | 0 | 0 | 0 |
| Shake hands | Left | Subject 5 | 0 | 0 | 0 |
| Write | Both arms | Subject 5 | 1 | 1 | 0 |
| Turn pages of a book | Both arms | Subject 5 | 1 | 2 | 1 |
| Use manual wheelchair | Both arms | Subject 5 | n.a. | n.a. | n.a. |
| Use keyboard of a computer | Both arms | Subject 6 | 2 | 2 | 0 |
| Eat with knife and fork | Both arms | Subject 6 | 2 | 2 | 0 |
| Shake hands | Right | Subject 6 | 1 | 2 | 1 |
| Shake hands | Left | Subject 6 | 1 | 2 | 1 |
| Write | Both arms | Subject 6 | 2 | 2 | 0 |
| Turn pages of a book | Both arms | Subject 6 | 2 | 2 | 0 |
| Use manual wheelchair | Both arms | Subject 6 | n.a. | n.a. | n.a. |

n.a. = not applicable because subject does not use a manual wheelchair
